# New Insights in Microplastic Cellular Uptake Through a Cell-Based Organotypic Rainbow-Trout (*Oncorhynchus mykiss*) Intestinal Platform

**DOI:** 10.3390/cells14010044

**Published:** 2025-01-03

**Authors:** Nicole Verdile, Nico Cattaneo, Federica Camin, Matteo Zarantoniello, Federico Conti, Gloriana Cardinaletti, Tiziana A. L. Brevini, Ike Olivotto, Fulvio Gandolfi

**Affiliations:** 1 Department of Veterinary Medicine and Animal Sciences, University of Milan, 26900 Lodi, Italy; nicole.verdile@unimi.it (N.V.); tiziana.brevini@unimi.it (T.A.L.B.); 2 Department of Life and Environmental Sciences, Marche Polytechnic University, 60131 Ancona, Italy; n.cattaneo@pm.univpm.it (N.C.); m.zarantoniello@univpm.it (M.Z.); f.conti@pm.univpm.it (F.C.); 3 Department of Agricultural and Environmental Sciences, University of Milan, 20133 Milan, Italy; federica.camin@unimi.it; 4 Department of Agricultural, Food, Environmental and Animal Sciences, University of Udine, 33100 Udine, Italy; gloriana.cardinaletti@uniud.it

**Keywords:** aquaculture, emerging pollutants, intestine, in vitro model, intestinal cells, organotypic platform, organoids

## Abstract

Microplastics (MPs) in fish can cross the intestinal barrier and are often bioaccumulated in several tissues, causing adverse effects. While the impacts of MPs on fish are well documented, the mechanisms of their cellular internalization remain unclear. A rainbow-trout (*Oncorhynchus mykiss*) intestinal platform, comprising proximal and distal intestinal epithelial cells cultured on an Alvetex scaffold, was exposed to 50 mg/L of MPs (size 1–5 µm) for 2, 4, and 6 h. MP uptake was faster in RTpi-MI compared to RTdi-MI. Exposure to microplastics compromised the cellular barrier integrity by disrupting the tight-junction protein zonula occludens-1, inducing significant decreases in the transepithelial-electrical-resistance (TEER) values. Consequently, MPs were internalized by cultured epithelial cells and fibroblasts. The expression of genes related to endocytosis (*cltca*, *cav1*), macropinocytosis (*rac1*), and tight junctions’ formation (*oclna*, *cldn3a*, *ZO-1*) was analyzed. No significant differences were observed in *cltca*, *oclna*, and *cldn3a* expression, while an upregulation of *cav1*, *rac1*, and *ZO-1* genes was detected, suggesting macropinocytosis as the route of internalization, since also *cav1* and *ZO-1* are indirectly related to this mechanism. The obtained results are consistent with data previously reported in vivo, confirming its validity for identifying MP internalization pathways. This could help to develop strategies to mitigate MP absorption through ingestion.

## 1. Introduction

Plastics are inexpensive, lightweight, and versatile materials that are frequently utilized in industries and for common purposes [1,2,3]. Plastic usage is increasing annually, and a growing trend is expected to continue in the next future [4]. Recent data demonstrated that in 2017, out of 9 billion tons of plastic produced, only 9% was recycled, allowing significant quantities of plastic to pollute the environment with detrimental effects on wildlife and humans [5]. In addition, the combined effect of physical, chemical, and biological processes often promotes plastic degradation into small plastic debris inevitably depositing in the natural environment [6]. When these debris reach a size smaller than 5 mm, they are categorized as microplastics (MPs), while particles smaller than 1 µm are classified as nanoplastics (NPs) [7].

MPs tend to accumulate in the aquatic habitats and consequently can be ingested by living organisms, posing serious concerns about their welfare [8,9]. Fish are particularly affected by MPs, along with other environmental contaminants, such as trace elements and organic micro-compounds, making them valuable bioindicators for assessing the presence of these pollutants in the environment [10,11,12,13,14]. Furthermore, MPs are inevitably introduced into the aquaculture systems mainly through the environment itself but also through aquafeeds [15], posing potential risks for the final consumers. Many fish species, both collected from the wild or from fish farms, have shown the presence of MPs in different tissues and organs [16,17,18]. In fact, the small size of MPs makes them easily ingestible by fish, posing significant risks to their welfare [19,20]. This facilitates trophic transfer and generates a plausible biomagnification at high trophic levels [21,22], raising serious concerns for human health as well [23,24,25].

Several studies have demonstrated that MP toxicity outcomes in fish are strictly related to the MP size, shape, chemical composition, and concentration [26,27,28,29,30], and when it comes to dietary MPs, it is obvious that the intestine plays a pivotal role in their assimilation. It has been shown that only MPs smaller than 20 µm are absorbed at fish intestinal level and consequently are able to cross the intestinal barrier, reaching other organs and tissues [31,32,33]. Furthermore, studies have shown that the intestine primarily serves as a transit organ for MPs, with the majority accumulating in the liver, although smaller amounts can also reach other organs [34,35,36]. MP ingestion and absorption lead to multiple toxic effects in fish, such as metabolic disorders, inflammation, morphological alteration of the intestinal mucosa, gut microbiota dysbiosis, oxidative stress responses, and an increase in the barrier permeability, resulting in a condition known as leaky gut [36,37,38,39,40,41,42]. Indeed, MPs can impair the proper intestinal barrier function by altering and disrupting the structural integrity of the epithelial junctional complexes, including tight and adherent junctions, both crucial for preserving the gut selective permeability [43,44]. Additionally, MPs have been also proposed to be actively absorbed at the cellular level through different mechanisms. Consequently, understanding the cellular mechanisms involved in MP uptake at the intestinal level is crucial for developing strategies to reduce their absorption, particularly in farmed fish. These efforts could improve fish welfare and reduce potential exposure to MPs in human consumers.

On this regard, despite that several molecular uptake mechanisms have been proposed to be crucial in the absorption of dietary MPs at intestinal level, including micropinocytosis, endocytosis (such as caveolin- and clathrin-mediated endocytosis), transcytosis, and paracellular diffusion, the knowledge related to this complex phenomenon in vivo is still fragmentary and largely unknown to such an extent that most uptake pathways are only hypothesized [45,46,47]. Moreover, while in vivo feeding trials are essential to assess the potential effects of MPs on the general animal welfare status, they are poorly useful for investigating the absorption pathways at the molecular and cellular levels [48,49]. Indeed, being an organism with a highly complex system, the correlation between the exposure to environmental contaminants, such as MPs, and an observed effect is not always immediate and could produce data of challenging interpretation [50,51]. Some drawbacks of in vivo studies can be overcome using in vitro tools, which, in some cases, are more suitable for carrying out investigations at the molecular and cellular levels [52,53]. In addition, these methods allow us to perform the experimentation in a tightly controlled environment with limited variations, minimizing costs and animal testing [54,55,56].

Recently, a 3D cell-based organotypic platform was developed [57], consisting of rainbow-trout (*Oncorhynchus mykiss*, Walbaum, 1792) epithelial and fibroblast cell lines cultured in combination with the synthetic scaffolding Alvetex™ (Reprocell, Orlando, FL, USA) a highly porous polystyrene insert that allows cellular infiltration and physiological interactions between neighboring cells. This advanced in vitro model replicates many of the morphological features of the native organ, with epithelial cells assuming a polarized phenotype and establishing an in vitro functional barrier, while fibroblasts generate a robust stroma, synthesizing an extracellular matrix and supporting the overhead epithelium [58]. Moreover, the platform proved to be suitable for functional nutritional tests, producing physiologically relevant responses when exposed to rainbow-trout (RT) aquafeeds [59,60]. Therefore, this tool should be a promising option for understanding the basic mechanisms related to MP assimilation in the intestine. In addition, although the fish gut morphology can vary according to the diet, habitat, and evolutionary history, the general structure of the intestinal wall at the cellular level is quite conserved among the fish species [61,62,63]. This conservation enables the extrapolation and application of the findings to several farmed fish species, facilitating the development of more targeted strategies aimed at reducing MP absorption at the cellular level.

The present study aimed to investigate the effects of MP exposure on an RT intestinal model to better understand MP uptake pathways.

## 2. Materials and Methods

### 2.1. Microplastic Features

Fluorescent MPs ranging from 1 to 5 µm (amino formaldehyde polymer, FMv-1.3; peak of emission at 636 nm when excited at 584 nm) were purchased from Cospheric LLC (Goleta, CA, USA). Before being resuspended in the cell culture medium, a 100× stock solution was prepared to dilute MPs in deionized water. The stock solution was mixed by vortexing and aliquoted into 2 mL tubes. Experimental doses were prepared by diluting the sterilized stock solution in cell culture medium.

### 2.2. Cell Lines

Two epithelial cell lines established from the proximal (RTpi-MI) and the distal (RTdi-MI) intestine of rainbow trout and a fibroblast cell line (RTskin01) derived from the trout dermis were used [64]. Epithelial cell lines were propagated in complete medium composed of Leibovitz’s culture medium (L-15, Thermo Fisher Scientific, cat. no. 11415064, Waltham, MA, USA) supplemented with 2 mM L-glutamine, 10,000 units/mL penicillin, 10.0 mg/mL streptomycin, 25.0 µg/mL amphotericin B (Merck, cat. no. A5955 Darmstadt, Germany), and 5% fetal bovine serum (FBS, Thermo Fisher Scientific, cat. no. 10270106, Waltham, MA, USA). RTskin01 fibroblasts were maintained in the same medium but supplemented with 10% fetal bovine serum (FBS). In all cases, rainbow-trout (RT) cell lines were grown at 20 °C under ambient atmosphere, expanded in 75 cm^2^ tissue culture flasks (T75, Sarstedt, cat. no. 83.3911, Nurmbrecht, Germany), and passaged at a 1:3 ratio, when reaching 95% confluency. The medium was replaced twice a week.

### 2.3. Preliminary Experiments on Plastic Surfaces

To exclude any toxic effects and to identify the most suitable MP concentration, RT epithelial cells were cultured on 24-well plates at the final density of 250.000 cells/cm^2^ for 24 h. Thereafter, rainbow-trout (RT) cells were exposed for 24 h to 3 increasing doses (12.5, 25 mg, and 50 mg/L) of fluorescent MPs. In addition, to investigate the mechanisms of internalization along time, three exposure times were tested (2, 4, and 6 h). Morphological changes were assessed with an inverted microscope. Evaluation of morphological changes after exposure was based on cell shape, eventual cell detachment, and presence of stress indicators, like granules or vacuoles.

### 2.4. Neutral Red Uptake (NRU) Assay

Lysosomal integrity after MP exposure was measured through the neutral red uptake (NRU) assay following the manufacturer’s indications (Sigma-Aldrich, cat. no. N2889-220ML, Darmstadt, Germany). Briefly, cells were washed in phosphate-buffered saline solution (PBS) and incubated for 1 h in the dark at 20 °C with the neutral red working solution (5.9 mL of PBS supplemented with 90 µL of neutral red). Then, they were washed in a fixative solution, consisting of 5 mM of CaCl_2_ in 10 mL of distilled water supplemented with 67 µL of neutral-buffered formalin, and incubated with an extraction solution (1:1 dilution of 96% ethanol and 2% glacial acetic acid) for 10 min under agitation. The supernatant was collected in a transparent flat-bottom 96-well plate and read at 540 nm using a Bio-Rad 680 (Hercules, CA, USA) microplate reader.

### 2.5. Estimation of MP Internalization

To determine MP internalization when cells were cultured onto a plastic surface, the F-actin staining with phalloidin, which defines the structure of the cell cytoskeleton, was combined with the staining of nuclei with 4′,6-diamidino-2-phenylindole (DAPI). Phalloidin-iFluor 594 (Abcam, cat. no. AB176757, Cambridge, UK) was diluted 1:1000 in PBS, and images were acquired using an Eclipse TE200 microscope (Nikon, Tokyo, Japan). After exposure to the different MP doses for 2, 4, and 6 h, cells were fixed in 4% paraformaldehyde (PFA) in PBS for 30 min at room temperature and washed thrice. Five representative images per sample were collected and, for each picture, the number of MP particles overlapping with the cytoskeleton was counted using the ImageJ v1.54 software and then divided by the number of nuclei.

### 2.6. Immunostaining for Zonula Occludens-1 (ZO-1)

To evaluate whether MP exposure would compromise the tightness of the rainbow-trout (RT) intestinal epithelial junctional complexes, the presence of intact zonula occludens was determined by immunofluorescence. In brief, cells cultured on 24-well plates and exposed for 2, 4, or 6 h to 50 mg/L were fixed in 4% PFA solution in PBS for 30 min at room temperature and washed thrice. Subsequently, aspecific bindings were prevented by incubating cells in 5% bovine serum albumin and 0.3% Triton X-100 in PBS for another 30 min. Samples were then incubated with a FITC conjugated anti-ZO-1 antibody diluted 1:100 in PBS (Life Technologies, cat. no. 339188, Waltham, MA, USA) for 1 h at room temperature. Nuclei were counterstained with DAPI for 20 min. Results of ZO-1 immunofluorescence were analyzed by applying the semi-quantitative scoring system described in Table 1 that we previously developed [60]. Briefly, 5 pictures were acquired for each sample. The ImageJ v1.54 software was used to transform images into 8-bit data (TIFF format), and a threshold was applied to discriminate the background from the specific signal of the immunostaining. The same threshold was applied for all samples.

### 2.7. Cell-Based Organotypic Platform Assembling

The cell-based organotypic platform was assembled as recently described [57]. Briefly, RTskin01 fibroblasts (10^6^ cells/well) were seeded into the highly porous polystyrene insert Alvetex™ (AV, Reprocell, cat. no. AVP005-12 Orlando, FL, USA). The same number of fibroblasts was added at days 7 and 9 of culture. Ascorbic acid (Sigma Aldrich, A4544-100G, 100 μg/mL, Darmstadt, Germany) was supplemented to the culture medium to promote collagen synthesis. After 14 days of culture, 9 × 10^5^ cell/well of RTpi-MI or RTdi-MI epithelial cells were layered on the top of the inserts and cultured for 21 days in complete medium without the ascorbic acid supplementation.

### 2.8. Establishment of an Effective Intestinal Barrier In Vitro

To check the formation of an effective intestinal barrier in vitro, transepithelial electrical resistance (TEER) was constantly monitored after seeding the epithelial cells. Measurements were performed using an EVOM2 epithelial voltmeter (World Precision Instruments, Berlin, Germany) equipped with an STX2 electrode as recently described [57]. Cells were exposed to MPs only after TEER value reached the plateau, indicating the formation of a functional epithelial barrier. To evaluate the eventual damage induced by MP exposure, TEER was measured after 2, 4, and 6 h of MP exposure. Controls were performed measuring TEER in samples not exposed to MPs and cultured with cell medium only.

### 2.9. MP Exposure and Evaluation of Cellular Response

**Figure 1:**
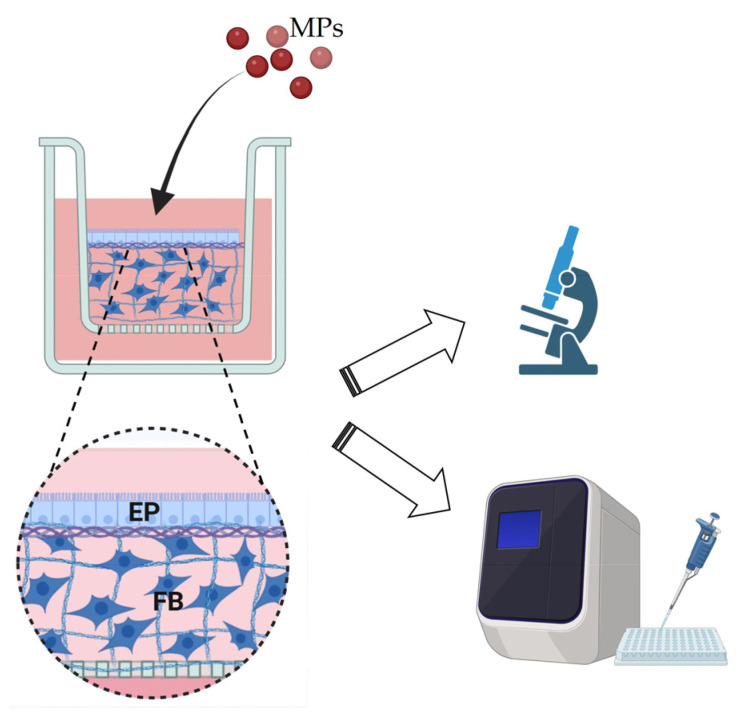
Graphical representation of the rainbow-trout-intestinal-platform organization (EP: epithelial cells, FB: fibroblasts) and experimental design. The intestinal platform was exposed to microplastics (MPs), and their effects were evaluated through morphological and molecular analysis (image was created using biorender.com).

Rainbow-trout proximal or distal (RTpi-MI or RTdi-MI, respectively) intestinal epithelial cells cultured on the AV platform were exposed to 50 mg/L for 2, 4, and 6 h. The spent medium was removed from the apical compartment of the bicameral inserts and replaced with fresh medium supplemented with fluorescent MPs. Culture medium with no MPs was used as negative controls. The effects of MP administration on cellular health and the relative uptake mechanisms were explored through morphological and molecular analysis (Figure 1).

After the trial, three samples grown on the Alvetex™ scaffolds were collected for each group (in triplicate; nine per experimental group). Each scaffold was divided into two halves, in which one was used for histology and the other for molecular analyses.

### 2.10. Histology

Samples for histology were fixed in 4% paraformaldehyde (PFA) overnight at 4 °C, dehydrated, cleared in Histoclear (Histo-Line laboratories, cat. no. R0050CITRO Pantigliate, Italy), and embedded in paraffin. Thin sections of 5 μm in thickness were stained with hematoxylin and eosin or with 4′,6-diamidino-2-phenylindole (DAPI) to assess the sample general morphology and to evaluate MP distribution in the AV scaffolding.

### 2.11. Evaluation of MP Distribution with Confocal Microscopy

Samples were fixed in PFA 4% for 24 h at 4 °C. Samples were then washed 3 times with a PBS and stocked in the same solution until further processing. Subsequently, samples were mounted on concave glass slides using a glycerol–PBS solution (90:10 ratio) and covered with a glass coverslip. The presence of fluorescent MPs in the samples was examined using a Nikon A1R confocal microscope (Nikon Corporation, Tokyo, Japan). Samples were excited with wavelengths of 561 and 647 nm at the same time, and the emissions were collected at 615 and 670 nm to visualize the MPs (in red) and cell nuclei (in blue), respectively. Image analysis was performed using the NIS-Element software (version 5.21.00; Nikon).

### 2.12. Molecular Analyses

After collection, samples were stored at −80 °C until further procedures. Total RNA was extracted with RNAzol™ (Merck) and eluted in 20 μL of RNase-free water (Qiagen, Hilden, Germany). DNase treatment (10 IU at 37 °C for 10 min, MBI Fermentas, Milano, Italy) was applied on total RNA to digest genomic DNA. The final concentration and integrity of RNA were assessed using a NanoPhotometer P-Class (Implen, München, Germany) and by electrophoresis of 1 μg of total RNA stained with GelRed^TM^ on a 1% agarose gel, respectively. RNA samples were stored at −80 °C. Subsequently, cDNA synthesis was performed on 1 μg of RNA using the iScript™ cDNA Synthesis Kit (Bio-Rad, Hercules, CA, USA) following the manufacturer’s instructions.

Real-time quantitative PCR (qPCR) was conducted using an iQ5 iCycler thermal cycler (Bio-Rad). Each reaction mixture comprised 1 µL of 1:10 diluted cDNA, 5 µL of fluorescent intercalating agent (2× concentrated iQ™ Sybr Green, Bio-Rad, Milano, Italy), and 0.3 µM of forward and reverse primers. The thermal cycle profile included an initial denaturation step at 95 °C for 3 min, followed by 45 cycles of denaturation at 95 °C for 20 s, annealing at the specific temperature for each primer for 20 s, and extension at 72 °C for 20 s. The annealing temperature for each primer was optimized using a temperature gradient assay. Primer specificity was confirmed by the absence of primer-dimer formation and dissociation curves. Additionally, primer efficiencies were assessed using a mix of cDNA (control group) with efficiencies around 90% for all primers and R^2^ values ranging from 0.995 to 0.998 at different concentrations (1:1, 1:10, 1:100, 1:1000). Fluorescence was monitored at the end of each cycle, and a single peak was observed for each qPCR product in the melting curve analyses. For each reaction, two no-template controls (NTCs) were included in every run to ensure the absence of contamination (no peaks were observed for the NTCs in any reaction). Amplification products were sequenced, and their homology was confirmed. The relative quantification of two genes associated with pinocytosis uptake was performed: clathrin heavy chain a (*cltca*), involved in clathrin-mediated endocytosis, and caveolin 1 (*cav1*), which plays a role in caveolin-mediated endocytosis. For macropinocytosis, *rac1* coding for a small GTP-binding protein was analyzed, while for cellular junction formation occludin a (*oclna*), claudin a (*cldn3a*) and zonula occludens-1 (*ZO-1*) were amplified. The primer sequences utilized in this study are provided in Table 2. The sequences were either obtained from previous studies or specifically designed using the NCBI Primer-BLAST tool, based on RT sequences available in GenBank. Internal reference genes, beta-actin (*b-actin*), and 60S ribosomal protein L31 (*rl31*), were used to standardize the results using the geometric mean of their expression levels following verification of their stable expression using algorithms integrated into the Bio-Rad CFX Manager 3.1 software. Changes in gene transcript expression levels among experimental groups are presented as relative mRNA abundance (in arbitrary units), following the methodology described in a previous study [65]. The qPCR data were processed using the iQ5 optical system software version 2.0 (Bio-Rad), along with the incorporation of the GeneEx Macro iQ5 Conversion and GeneEx Macro iQ5 files.

### 2.13. Statistical Analysis

All data were checked for normality using the Shapiro–Wilk test, and the homogeneity of variances was verified using Levene’s test. Since, in all cases, the *p* value was >0.05, statistical analyses were performed using one-way analysis of variance (ANOVA), followed by Tukey’s multiple comparison post hoc test, utilizing the Prism 8 software (GraphPad, version 8.0.2, San Diego, CA, USA). Statistical significance was defined as the *p* value < 0.05. In the graphs, the different letters above the columns (a, b, and c) indicate statistically significant differences among experimental groups, whereas “ns” denotes no significant difference.

## 3. Results

### 3.1. Cell Morphology and Viability

Before exposing the complex 3D cell-based organotypic intestinal model to MPs, preliminary tests on cells cultured onto a simple plastic surface were performed to (i) exclude any MP toxic effect, (ii) verify MP uptake, (iii) identify the most suitable MP concentration, and (iv) identify the most appropriate time of exposure.

**Figure 2:**
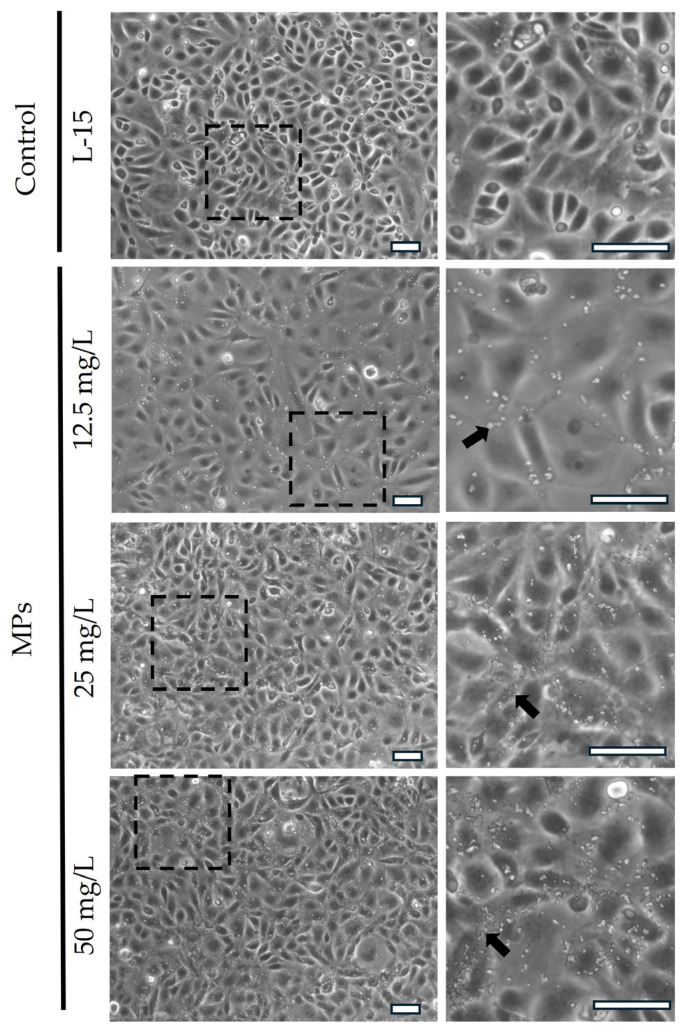
Representative brightfield microscopy images showing RTdi-MI epithelial cells exposed to MPs. The presence of MPs (arrows) in the treated samples at different concentrations. No MPs were detected in the control samples, cultured with the cell culture medium only (scale bar 50 µm).

Brightfield microscopy images showed the presence of MPs in the treated samples, and, as expected, no MPs were visible in the respective control. A 24 h exposure to MPs did not affect cell morphology, which was comparable to the control (L-15 medium) even at the highest concentration (Figure 2).

**Figure 3:**
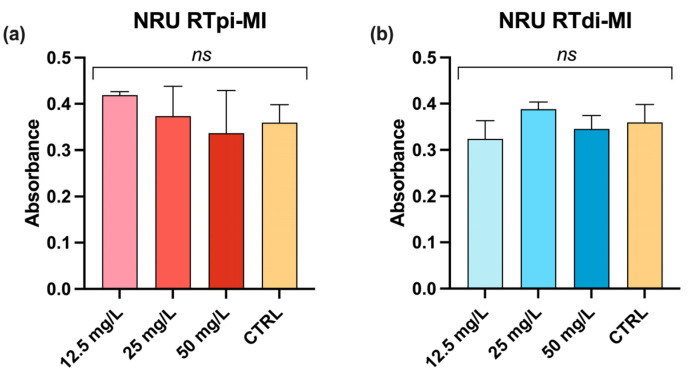
Neutral red uptake (NRU) assay showing cell viability after exposure to increasingly higher concentrations of MPs in proximal (RTpi-MI) (**a**) and distal (RTdi-MI) (**b**) intestinal rainbow-trout (RT) cell lines for 24 h. Controls (CTRLs) were performed by measuring the cell viability of RT cell lines cultured with medium only. Values are expressed as mean ± standard deviation (ns, indicates no statistically significant differences (RTpi-MI: *p* = 0.47, F = 0.92; RTdi-MI: *p* = 0.17, F = 2.22, *n* = 3), determined by one-way ANOVA).

Moreover, the neutral red uptake (NRU) assay showed non-significant differences among treated and control cells (CTRL) regardless of the tested concentrations, indicating that MPs were not toxic to the cells (Figure 3).

### 3.2. MP Internalization

**Figure 4:**
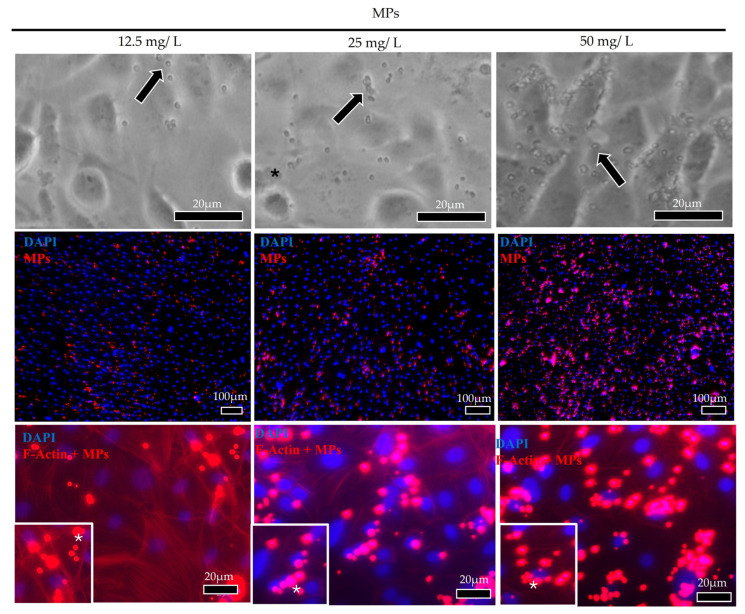
Representative brightfields and F-actin images (arrows and red signal) showing MP (red dot/asterisks) internalization in cell cytoplasm of the rainbow-trout proximal intestinal (RTpi-MI) epithelial cells. MP internalization followed a dose-dependent pattern, being the highest when 50 mg/L were exposed. MPs were mainly distributed in the perinuclear region (nuclei were stained with DAPI—blue signal).

**Figure 5:**
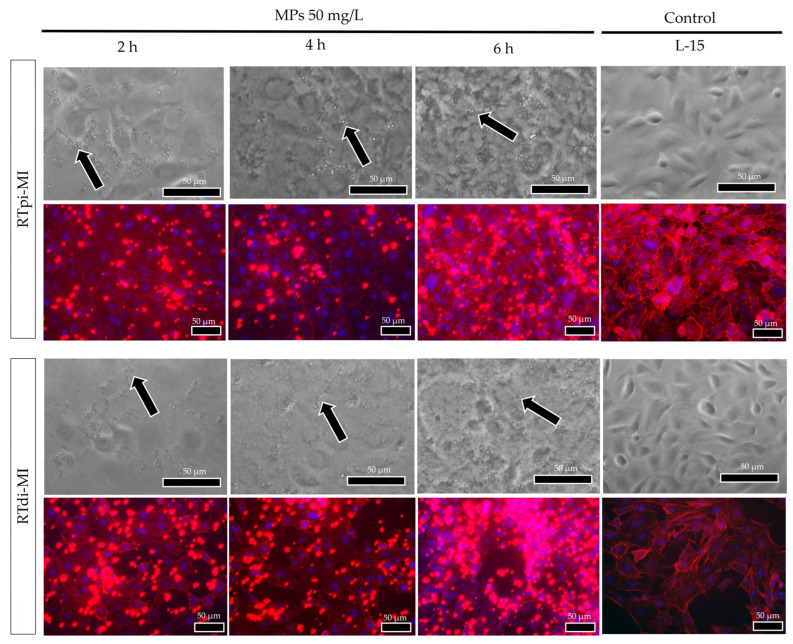
Representative brightfields and F-actin (red signal) images showing MP (arrows and red dots) internalization in cell cytoplasm of the treated samples after 2, 4, and 6 h to the highest MP concentrations (50 mg/L) for both rainbow-trout cell lines (RTpi-MI and RTdi-MI). No differences were observed between the two cell lines. (Nuclei were stained with DAPI—blue signal).

F-actin combined with DAPI staining showed that MPs were mainly distributed in the proximity of cell nuclei and that MP internalization within the cell cytoplasm followed a dose-dependent pattern, being highest after exposure to a MP concentration of 50 mg/L (Figure 4), with no differences between the two cell lines (Figure 5).

**Figure 6:**
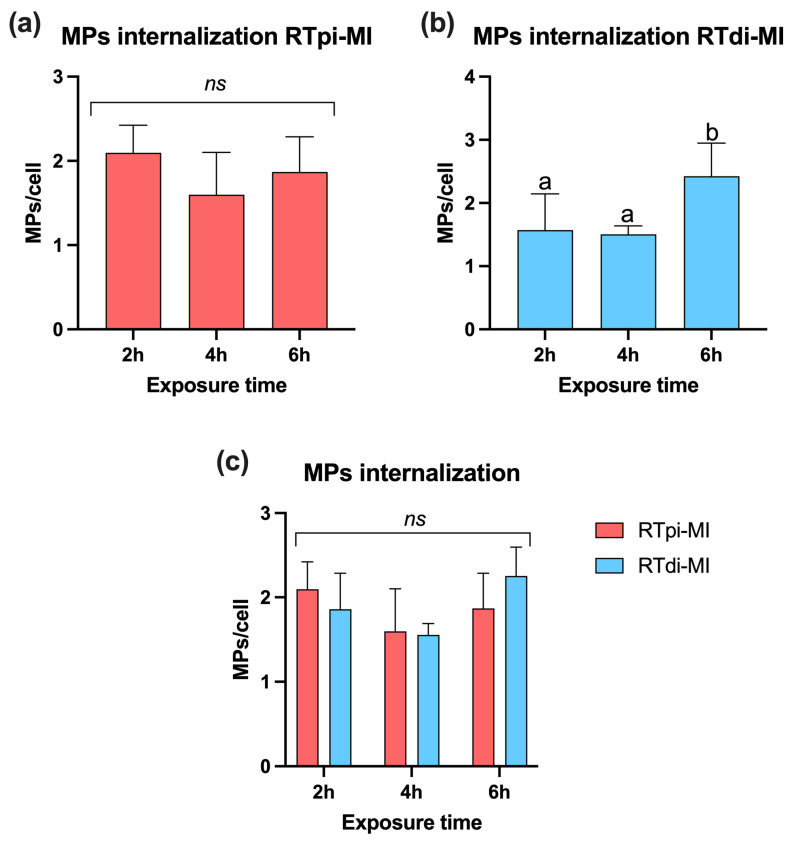
Bar charts showing quantification of MP internalization at the highest concentration (50 mg/L) in proximal (RTpi-MI) and distal (RTdi-MI) cell lines after 2, 4, and 6 h of exposure. (**a**) RTpi-MI; (**b**) RTdi-MI; (**c**) RTpi-MI and RTdi-Mi comparison. Values are expressed as mean ± standard deviation (in each graph, different letters indicate significant differences *p* < 0.05; ns denotes no significant differences among the exposure time *p* > 0.05, *n* = 3). Statistical differences were determined by one-way ANOVA ((**a**): RTpi-MI: *p* = 0.29, F = 1,45; (**b**) RTdi-MI: *p* < 0.5, F = 6.96; (**c**) *p* = 0.08, F = 2.37).

After 2, 4, and 6 h of exposure to 50 mg/L of MPs, no significant differences were observed in the amount of MP absorption per cell in the proximal cell line (RTpi-MI; Figure 6a) (*p* = 0.29). Conversely, in distal cells (RTdi-MI; Figure 6b), the MP uptake was significantly higher (*p* < 0.05) after 6 h of exposure compared to 2 and 4 h. However, comparing MP internalization after 2, 4, and 6 h between the two cell lines, no statistically significant differences (*p* = 0.08) were observed (Figure 6c).

### 3.3. Zonula Occludens-1 (ZO-1) Immunostaining

**Figure 7:**
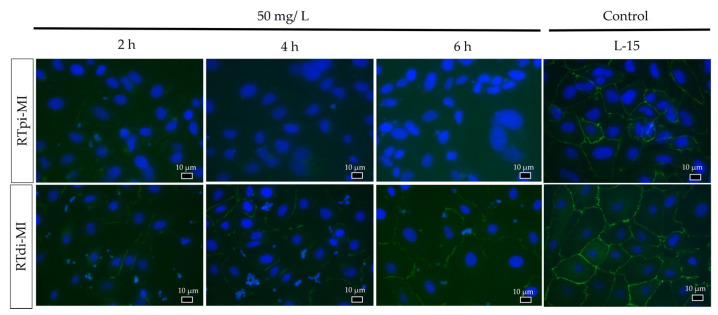
Representative images of ZO-1 immunostaining (green signal), showing a specific and clear signal in the controls (rainbow-trout proximal and distal intestinal cells not exposed to MPs) and a weak and fragmented signal in samples exposed to MPs (50 mg/L). (Nuclei are stained with DAPI—blue signal.) (RTpi-MI: rainbow-trout proximal cell line, RTdi-MI: rainbow-trout distal intestinal cell line).

**Figure 8:**
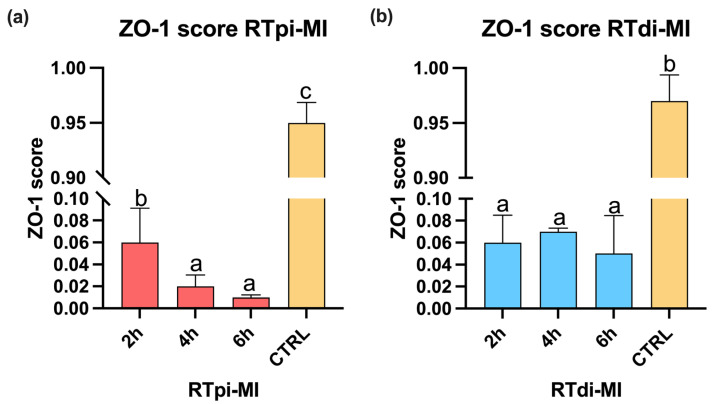
Bar charts showing ZO-1 score in (**a**) RTpi-MI (proximal) and (**b**) RTdi-MI (distal) cell lines after 2, 4, and 6 h of exposure to the highest MP concentration (50 mg/L). CTRL represents the control cell line not exposed to MPs. Values are expressed as mean ± standard deviation. Different letters in each graph indicate significant differences (*p* < 0.05, *n* = 3). Statistical differences were determined by one-way ANOVA ((**a**): RTpi-MI: *p* < 0.05, F = 3573; (**b**) RTdi-MI: *p* < 0.05, F = 2085).

While in the samples not exposed to MPs (controls), immunostaining showed a clear and specific signal for ZO-1 in both cell lines, and the signal appeared weak and strongly fragmented in the experimental conditions. In particular, no *signal was detected* in most proximal epithelial cells (RTpi-MI) (Figure 7). However, the semi-quantitative scoring system highlighted that in both lines after 2 h of MPs exposure, ZO-1 was already significantly compromised compared to the respective controls. However, while in the distal cell line (RTdi-MI), the damage was stable along the 6 h of exposure (Figure 8b), in the proximal cell line (RTpi-MI), the damage significantly worsened after 4 h of exposure (Figure 8a).

Since no differences (*p* > 0.05) were observed among the different dosages used in terms of morphology, cell viability, and MP uptake and considering that 50 mg/L represents a concentration similar to that found in contaminated feed [69], this concentration was selected for the experiments using the complex 3D model.

### 3.4. Measurements of the Transepithelial Electrical Resistance (TEER)

**Figure 9:**
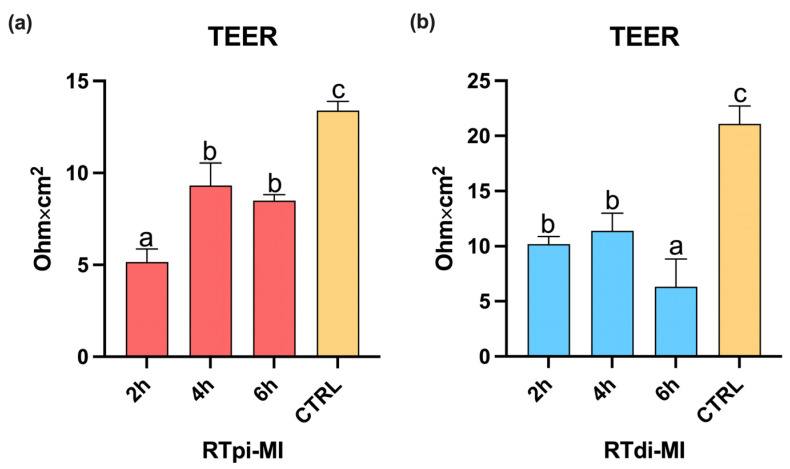
Bar charts showing TEER measurements in (**a**) RTpi-MI (proximal) and (**b**) RTdi-MI (distal) cell lines after 2, 4, and 6 h of exposure to the highest MP concentration (50 mg/L). CTRL represents the control cell line not exposed to MPs. Values are expressed as mean ± standard deviation (*n* = 9). Different letters in the same graph indicate statistically significant differences (*p* < 0.05). Statistical differences were determined by one-way ANOVA ((**a**): RTpi-MI: *p* < 0.05, F = 117; (**b**) RTdi-MI: *p* < 0.05, F = 78.40).

In both cell lines, exposure to MPs resulted in a significant reduction in TEER values compared to the controls (CTRLs) after 2 h of exposure. Unexpectedly, after 4 and 6 h of exposure, TEER values were partially recovered in RTpi-MI (proximal cell line; Figure 9a). Conversely, in RTdi-MI (distal cell line), TEER values further significantly decreased after 6 h of exposure (Figure 9b).

### 3.5. MP Migration Throught the 3D Scaffold

**Figure 10:**
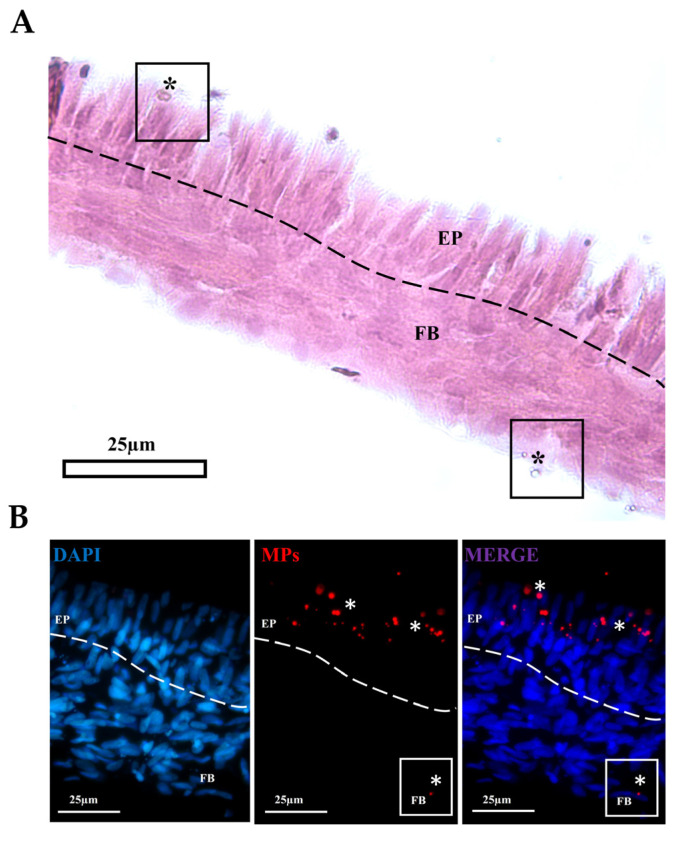
Representative hematoxylin-eosin- (**A**) and DAPI-stained section (**B**) showing rainbow-trout proximal cells exposed to MPs (asterisks) for 2 h. MPs are absorbed by epithelial cells (EP) and cross the barrier reaching the stroma, where a few are internalized also by fibroblasts (FB). Dotted line represents the boundary between the connective tissue and the overhead epithelium.

As expected, both intestinal epithelial cell lines grown on the AV platform formed a monolayer of cubic cells on top of a supportive layer consisting of fibroblasts and collagen. Light microscopy showed that, after a 2 h exposure, MPs not only penetrated into the epithelial cells but also crossed the barrier and reached the stroma (Figure 10).

### 3.6. Confocal Microscopy

**Figure 11:**
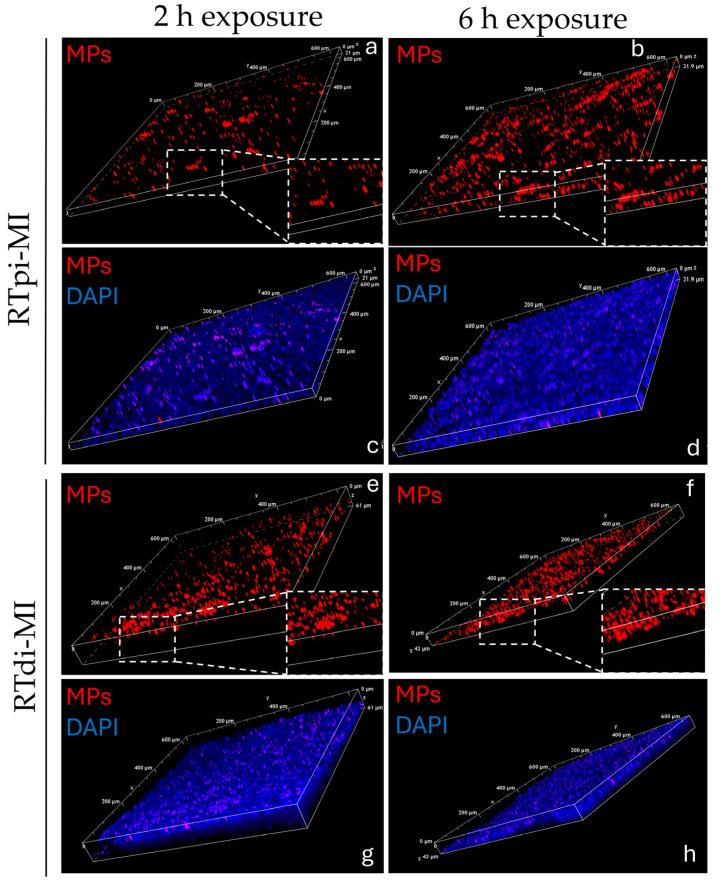
Three-dimensional z-stack sections of the rainbow-trout proximal and distal cell lines (RTpi-MI and RTdi-MI, respectively). Representative images of (**a**–**d**) RTpi-MI cell lines and (**e**–**h**) RTdi-MI cell lines. Different time of exposure to MPs sized 1–5 µm at 50 mg/L: after 2 h; after 6 h. Nuclei are counterstained with DAPI (blue signal). Red dots indicates fluorescent MP beads (size 1–5 µm). (RTpi-MI: rainbow-trout proximal intestinal cell line, RTdi-MI: rainbow-trout distal intestinal cell line).

Analysis by confocal microscopy of both cell lines (RTpi-MI and RTdi-MI) confirmed the presence of fluorescent beads within the epithelial cells (Figure 11). After 2 h exposure, MPs crossed the epithelial barrier in both lines (Figure 11a,c). At 6 h, in the proximal intestinal line (RTpi-MI), all the MPs reached the basal cells (Figure 11b), while in the distal intestinal line (RTdi-MI), some of them were still crossing the membrane (Figure 11d).

### 3.7. Gene Expression Analysis

**Figure 12:**
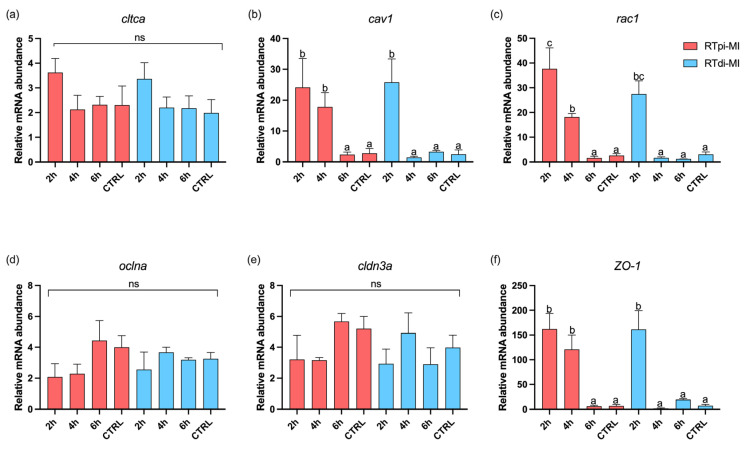
Relative mRNA abundance of genes involved in intracellular uptake (*cltca*, *cav1*, and *rac1*) and cellular junction formation (*oclna*, *cldn3a*, and *ZO-1*) analyzed in the membrane of the two rainbow-trout intestinal cell lines (RTpi-MI: proximal intestinal line; RTdi-MI: distal intestinal line) after 2 h, 4 h, and 6 h of exposure to 50 mg/L of MPs (size 1–5 µm). CTRL represents the control cell line not exposed to MPs. (**a**) *cltca*, clathrin heavy chain a; (**b**) *cav1*, caveolin 1; (**c**) *rac1*, small GTP-binding protein; (**d**) *oclna*, occludin a; (**e**) *cldn3a*, claudin a; (**f**) *ZO-1*, zonula occludens-1. In each graph, different letters denote significant differences among the experimental groups. Data are reported as mean ± SD (*n* = 9). (ns, no significant differences among the experimental groups (*p* > 0.05)). Statistical differences were determined by one-way ANOVA ((**a**) *cltca*: *p* = 0.08, F = 3.73; (**b**) *cav1*: *p* < 0.05, F = 13.40; (**c**) *rac1*: *p* < 0.05, F = 39.40; (**d**) *oclna*: *p* = 0.07, F = 3.05; (**e**) *cldn3a*: *p* = 0.06, F = 3.43; (**f**) *ZO-1*: *p* < 0.05, F = 32.23).

With regards to the genes involved in intracellular uptake when RT cells were cultured in a 3D setting, no significant difference was observed among the experimental groups and the control groups in the expression of the *cltca* gene associated with clathrin-mediated endocytosis (Figure 12a). On the contrary, *cav1*, the gene related to caveolin-mediated endocytosis, exhibited a significant (*p* < 0.05) upregulation in proximal cells (RTpi-MI) at 2 h and 4 h and, in distal cells (RTdi-MI), at 2 h compared to the others (Figure 12b). A similar expression pattern was found for the *rac1* gene, which encodes the small GTP-binding protein, with the difference that the expression in RTpi-MI at 4 h was significantly lower (*p* < 0.05) than in RTpi-MI at 2 h (Figure 12c).

The relative expression of *oclna* and *cldn3a* genes, both involved in cellular junction formation, showed no difference (*p* > 0.05) among the experimental and the control groups (Figure 12d,e). However, the *ZO-1* gene, that encodes for the zonula occludens-1 protein, showed a significant (*p* < 0.05) upregulation in RTpi-MI at 2 h and 4 h, as well as in RTdi-MI at 2 h, compared to the other groups (Figure 12f), following the same pattern observed for *cav1* (Figure 12b).

## 4. Discussion

Currently, the in vivo understanding of the MP internalization, despite several proposed uptake mechanisms such as endocytosis, transcytosis, and paracellular diffusion, remains fragmented and largely hypothetical [46]. In this context, emerging evidence suggests that advanced in vitro models of the gut, designed to faithfully replicate the intestinal mucosa, can serve as robust tools to investigate the effects and uptake mechanisms of MPs on the intestinal epithelial barrier under various stimuli [70,71]. Therefore, here, several intercellular and extracellular MP internalization pathways were analyzed together with the key proteins involved in preserving the barrier integrity.

Experiments conducted on 2D supports have been used as preliminary screening tools before transitioning to more complex 3D platforms. This approach allowed us to obtain rapid results and to identify optimal doses, timing, and internalization patterns that deserved further investigation in more complex 3D models. A total of 24 h of exposure to MPs cultured on standard 2D support did not affect cell morphology even at the highest concentrations used in this study, confirming recent results obtained exposing another rainbow-trout cell line (RTgutGC cells) to polyethylene MPs [72]. Moreover, exposing RT epithelial cells to MPs at any dose did not decrease cell viability, indicating no short-term toxicity. This observation contrasts with a previous study describing that cryogenically milled tire tread particles, used as a proxy for tire and road wear particles, had a toxic effect on cell lines representing the gill (RTgill-W1) and the intestinal (RTgutGC) epithelium only at doses largely exceeding those found in the environment [73], possibly due to differences in polymers and pre-exposure treatments. In line with this, in vivo studies have shown that MP ingestion can lead to polymer- and species-specific outcomes [27,74]. However, similar to the findings of the present study, other research on multiple cell lines treated with MPs of 200 nm to 10 µm, and on Caco-2 cells exposed to 2 µm MPs, found no detrimental effects on cell viability [75,76]. Additionally, a lipid-membrane-model study showed that MPs sized 1–10 µm could induce mechanical stress, potentially activating proteins involved in MP internalization [77].

Filamentous actin, being a crucial component of the cell cytoskeleton, actively participates in particles’ internalization. In this study, F-actin staining was used to detect internalized MPs when cells were cultured on plastic surfaces. In both cell lines, MPs translocate from the extracellular space into the cell cytoplasm, confirming that trout intestinal cell lines are suitable for ecotoxicological studies, consistent with a previous study [72]. While in RTpi-MI (proximal cell line), MPs were completely internalized after 2 h of exposure, in RTdi-MI (distal cell line), their absorption occurred more slowly and was at a maximum after 6 h, indicating a sort of resistance to their internalization. Interestingly, in vivo, the two intestinal portions exert different functions. While the proximal intestine is responsible for about 70% of nutrient absorption, the distal intestine represents the major immunological district [78]. Therefore, it is plausible to hypothesize that the proximal cell line could be the most susceptible also to MP internalization. Furthermore, the fact that MPs were preferentially distributed in the perinuclear region suggests their accumulation in the endoplasmic reticulum. This is consistent with the recent observation that polystyrene MPs induce endoplasmic reticulum stress in the kidney of juvenile rats [79] and in the carp intestine by activating caspase-associated genes [80].

Analyzing the expression of the genes involved in cellular uptake, when cells were cultured in a 3D setting, no difference was detected among experimental groups for clathrin heavy chain A (*cltca*), which is involved in clathrin-mediated endocytosis. This can be explained by the fact that *cltca* forms vesicles with a maximum size of 200 nm [81], which are too small to include the MPs used in this study (size 1–5 µm). Two distinct studies support this hypothesis, demonstrating that the absorption of 50 nm MPs by human intestinal organoids and by a cell membrane model was clathrin-mediated endocytosis [82,83]. On the contrary, the expression of the *cav1* gene was significantly higher than that of controls in the proximal line (RTpi-MI) at 2 and 4 h, and in the distal line (RTdi-MI) at 2 h, suggesting a MP internalization through caveolin-mediated endocytosis. In fact, it has recently been demonstrated that caveolin-1 is not only implicated in the genesis of caveolae vesicles (size range 50–100 nm) [84,85] but also in the formation of much larger extracellular vesicles [86] (ranging from 100 to 1000 nm), as well as of exosomes (size 30–150 nm), implicated in inflammation, immune modulation, and cell communication [87].

Even if the smaller MPs of 1 µm could be transported by the extracellular vesicles, alternative absorption mechanisms for the MP sizes investigated in this study should be considered. In particular, it was interesting to find that the expression of *rac1*, a gene involved in micropinocytosis, encoding proteins responsible for the formation of macropinosomes, large vesicles ranging between 0.2 to 5 µm in diameter [88], was significantly higher than in controls at 2 and 4 h in RTpi-MI and at 2 h in RTdi-MI. Additionally, the expression in RTpi-MI at 4 h was significantly lower than at 2 h. This data is consistent with the observation that most MPs were internalized by these cells within the first two hours of exposure; therefore, it suggests that the mechanism involved in the cellular uptake of MPs sized 1–5 µm is likely macropinocytosis. This result is consistent with numerous studies conducted on different cellular models. For instance, studies on mouse macrophages, Caco-2, and various other cell lines have all demonstrated that MPs ranging in size from 1 to 10 µm are internalized via both macropinocitosys and phagocytosis [89,90,91]. However, even if generally there is a positive correlation between mRNA and proteins levels, it must considered that the mechanisms occurring during protein translation can be affected by several factors [92,93]. Unfortunately, working with a non-conventional species limited our capacity to detect changes in protein level due to the lack of specific reactive antibodies.

Intestinal junctional complexes are essential to maintain the barrier functions. In particular, zonula occludens proteins play a key role in preserving tight-junction integrity and in guiding cell proliferation and differentiation [94]. Being crucial to maintain epithelial cell sealing, ZO-1 damage correlates with a condition known as leaky gut [43]. This mechanism implies the loss of the intestinal selective permeability, allowing the paracellular flux of potential toxic substances [41]. In the current experiment, exposure to MPs induced ZO-1 protein disruption on standard 2D support. This was especially evident in the proximal cell line (RTpi-MI), confirming its higher sensitivity compared to the distal one (RTdi-MI). Consistently, TEER values, measured when RT cells were exposed to MPs and were cultured in a 3D environment, significantly decrease further, indicating the perturbation of the integrity of the epithelial barrier. Analogous results were given by a study conducted on the Caco-2 cell line indicating that 100 nm MP particles can induce disruption of tight junctions [95]. Another study reported similar findings with 20 nm MPs and additionally observed downregulation of genes encoding tight-junction proteins (*ocln* and *cldn1*) when particles were administered at 100 and 1000 µg/mL (no difference was detected at lower concentrations) [76]. The expression pattern of these genes aligns with that observed in our experiment, where administration of 1–5 µm MPs at 50 µg/mL did not impair *oclna* and *cldn3a* expression. However, the expression of the gene *ZO-1* was significantly higher in the proximal line at 2 and 4 h and at 2 h in the distal line. This observation is in apparent contradiction with the pattern of ZO-1 protein localization described above, where a clear cell profile became fuzzy following exposure to MPs. This can be explained considering that the ZO-1 protein not only maintains the integrity of tight junctions but also interacts with signaling pathways involved in the regulation of macropinocytosis [96]. ZO-1 can influence the activity of small GTPases like Rac1, which are key regulators of actin cytoskeleton dynamics and macropinocytosis and are critical for the formation and progression of macropinosomes [97]. The higher expression of *ZO-1* in the MP-treated groups, therefore, could be related to the modulation of the gene *rac1* in the formation of macropinosomes and interaction with the macropinocytosis mechanism. This observation supports our hypothesis that MP particles ranging from 1 to 5 µm in size are internalized via macropinocytosis. Additionally, constitutive *rac1* expression has been reported to disrupt tight-junction organization, leading to the disassembly of tight-junction strands and altered protein distribution of key components, such as occludin, ZO-1, and actin [98]. At the same time, the fact that a functional epithelial barrier seems to be compromised by a faulty location of the ZO-1 protein along the cell’s apical border, especially in the proximal cell line, could further explain the faster MP internalization that we observed. In particular, this aspect could be related to previous observations in humans, where compromised tight-junction integrity results in a non-selective permeability pathway predisposing the organisms to several injurious events [99]. The cell-based organotypic platform used in this study includes not only a functional epithelial barrier but also a robust stroma made by fibroblast and collagen [57]. This enabled us to observe that already after 2 h of exposure, not only MPs are absorbed within the epithelial cells but cross the gut barrier and are absorbed also by fibroblasts. Therefore, our model replicates both the macropinocytotic pathway that leads to the internalization of MPs within the epithelial cells as well as the paracellular passage of MPs that end up in the connective stroma.

## 5. Conclusions

In conclusion, the rainbow-trout cell-based organotypic platform used in the present study has proved effective for assessing the absorption mechanisms of potential environmental contaminants like MPs. The fact that it replicates both the intestinal epithelium and the supporting connective tissue in vitro enabled us to identify two main MP uptake mechanisms: paracellular diffusion and macropinocytosis. In the future, the exposure of MPs could be combined with potential natural bioactive compounds, such as astaxanthin, glutamine, polyphenols, and vitamins, to explore efficient strategies to mitigate or prevent MP absorption at the intestinal level [100].

## Figures and Tables

**Table 1:** Semi-quantitative scoring system applied to evaluate *zonula occludens-1* (ZO-1) immunostaining.

Score	Descriptive Parameter
3	cells having an intact ZO-1
2	cells having at least 1/4 of ZO-1 without fragmentation
1	cells having a ZO-1 highly fragmented
0	cells without ZO-1

**Table 2:** Gene name, sequences, annealing temperatures (AT), source, and amplicon size of primers used in the present study.

Gene	Forward Primer (5′-3′)	Reverse Primer (5′-3′)	AT (°C)	Source	Amplicon Size
*cltca*	GGCTGTCCGTAACAATCTAGCTG	GCAGCCTCAGAGTAGTTTCCC	58	XM_036937421.1	90
*cav1*	GTGCTACCGTCTCCTCACTG	ACCGCCCAGATGTGAATGAA	59	XM_021576628.2	96
*rac1*	CAGCAGGACAGGAAGACTACG	ATCCAGCTTGGTGTCTCACCT	58	NM_001160673.1	147
*oclna*	TTTGGTGGTGCTGCCTATGG	GCCGTGATGAAGCTGAATGC	57	NM_01190446.1 [66]	125
*cldn3a*	GGATCATTGCCATCGTGTCCT	AACACAGGTCATCCACAGGC	59	BK007964.1 [66]	113
*ZO-1*	AAGGAAGGTCTGGAGGAAGG	CAGCTTGCCGTTGTAGAGG	58	HQ656020 [67]	291
*b-actin (hk)*	AGACCACCTTCAACTCCATCAT	AGAGGTGATCTCCTTCTGCATC	59	AJ438158.1 [68]	131
*rl31 (hk)*	TTCCTGTCACGACATACAAAGG	GTAAGCAGAAATTGCACCATCA	60	NM_001165047.2 [68]	157

## Data Availability

The data presented in this study are available on request from the corresponding author.
